# Surface Display of Recombinant *Drosophila melanogaster* Acetylcholinesterase for Detection of Organic Phosphorus and Carbamate Pesticides

**DOI:** 10.1371/journal.pone.0072986

**Published:** 2013-09-09

**Authors:** Jingquan Li, Qian Ba, Jun Yin, Songjie Wu, Fangfang Zhuan, Songci Xu, Junyang Li, Joelle K. Salazar, Wei Zhang, Hui Wang

**Affiliations:** 1 Key Laboratory of Food Safety Research, Institute for Nutritional Sciences, Shanghai Institutes for Biological Sciences, Chinese Academy of Sciences, Shanghai, P. R. China; 2 Institute for Food Safety and Health, Illinois Institute of Technology, Bedford Park, Illinois, United States of America; 3 Key Laboratory of Food Safety Risk Assessment, Ministry of Health, Beijing, P. R. China; Weizmann Institute of Science, Israel

## Abstract

Acetylcholinesterase (AChE) is commonly used for the detection of organophosphate (OP) and carbamate (CB) insecticides. However, the cost of this commercially available enzyme is high, making high-throughput insecticide detection improbable. In this study we constructed a new AChE yeast expression system in *Saccharomyces cerevisiae* for the expression of a highly reactive recombinant AChE originating from *Drosophila melanogaster* (DmAChE). Specifically, the coding sequence of DmAChE was fused with the 3′-terminal half of an α-agglutinin anchor region, along with an antigen tag for the detection of the recombinant protein. The target sequence was cloned into the yeast expression vector pYes-DEST52, and the signal peptide sequence was replaced with a glucoamylase secretion region for induced expression. The resultant engineered vector was transformed into *S. cerevisiae*. DmAChE was expressed and displayed on the cell surface after galactose induction. Our results showed that the recombinant protein displayed activity comparable to the commercial enzyme. We also detected different types of OP and CB insecticides through enzyme inhibition assays, with the expressed DmAChE showing high sensitivity. These results show the construction of a new yeast expression system for DmAChE, which can subsequently be used for detecting OP and CB insecticides with reduced economic costs.

## Introduction

Organophosphate (OP) and carbamate (CB) insecticides have been widely used in the agricultural industry to increase the yield of crops [1], [2]. These pesticides are applied to plant surfaces or the soil itself to kill target organisms, mainly insects. However, OP and CB insecticides have been shown to be harmful on the environment, agricultural workers, and the surrounding human and animal population [2]. It is known that the toxicity of OP and CB insecticides is mainly due to the inhibition of a key enzyme, acetylcholinesterase (AChE, EC3.1.1.7), that plays a role in neural conduction pathways [3]–[5]. AChE hydrolyzes the important neurotransmitter acetylcholine, which assures the normal conduction of nerve impulses [6]–[8]. OP and CB insecticides bind to AChE and inhibit the native hydrolytic ability through phosphorylating the serine residues in the hydroxyl group [8]. After AChE is inhibited, acetylcholine accumulates in the synapse and results in blocked neural conduction pathways [9]–[14].

Many detection methods are currently available as a measure of protection against the harmful effects of OP and OB insecticides. However, a novel system that would detect residual OP and CB insecticides quickly and accurately remains to be developed. The more routine detection methods are commonly based on spectrum and chromatographic techniques [15]–[18], which are costly and time-consuming due to complex experimental procedures. Recently, AChE-based analytical methods have gained increasing interest due to the high speed and sensitivity attributed to on-site detection. According to previous studies, insect-originated AChE is more sensitive and more suitable to be used for detection of OP and CB insecticides compared with mammal-originated AChE [19]. However, the experimental procedures for extraction and purification of AChE from insects are time consuming and costly. To overcome these problems, different expression systems have been used, such as vaculovirus, yeast, Xenopus, oocytes and mammalian cells [20]–[25]. Among them, the yeast expression systems have the advantage of high enzymatic yields and stable fermentation characteristics [22].

In the current yeast expression system, AChE is synthesized and secreted under certain conditions in a commercially available detection kit. AChE dry powder is commonly used due to its high stability and purity. However, the separation and purification of secreted enzyme is methodologically complex. Therefore, there is an urgent need to improve the existing yeast expression system to produce usable enzyme directly. The yeast surface display technique could very well be the appropriate solution. This technique has great potential in facilitating the production of sufficient usable quantities of functional AChE because yeast itself could be regarded as an immobilization carrier.

In the current study, we applied the surface display technique in the DmAChE yeast expression system and determined the production and activity of DmAChE. In our research, we fused the *ace* gene of *D. melanogaster* with a yeast α-agglutinin gene to allow expression of DmAChE on the surface of yeast [26]–[29]. After inducement and fermentation, the yeast suspension can be used directly for OP and CB insecticides detection, with the activity and sensitivity comparable to the commercial enzyme at a significantly lower cost.

## Results

### The identification of clones

**Figure 1:**
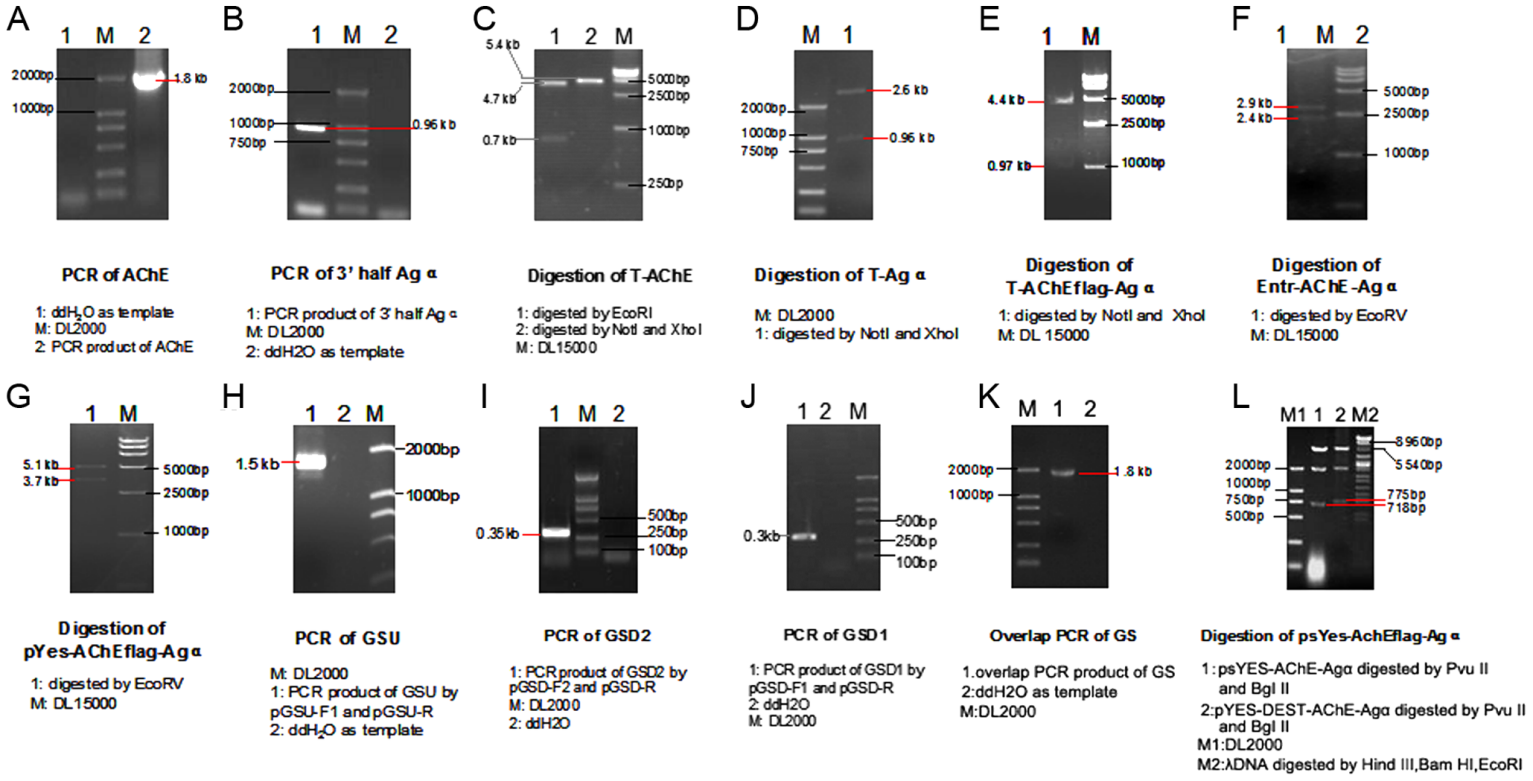
The identification of PCR constructs.

To ensure that the constructed vector contained the correct fragments, PCR analysis was used as verification during all construction steps. The fragments of DmAChE (1.8-kb) and the 3′ of Agα (0.9-kb) were obtained from *D. melanogaster* and *S. cerevisiae* respectively (Figure 1A and 1B, respectively). After digestion with appropriate restriction enzymes, the 4.7-kb and 0.7-kb fragments showed that the DmAChE fragment was inserted into pMD18-T accurately (Figure 1C), while the 0.96-kb and 2.6-kb fragments (Figure 1D) showed that the fragment was inserted into the 3′ of Agα. Figure 1E and 1F show that the FLAG region, together with the AChE-Agα fragments, were inserted into pMD18-T and pENTR™ Directional TOPO. After recombination with pYes-DEST52, the fragment containing DmAChE and the 3′ of Agα, along with the FLAG, was inserted into pYes-DEST52 (Figure 1G). The 1.4-kb GSU (Figure 1H) and the 0.4-kb GSD2 (Figure 1I), which was amplified by PCR using GSD1 (Figure 1J) as template, were ligated by overlap PCR to get GS (Figure 1K). GS was digested and ligated into the vector to get the final construct (Figure 1L).

### Optimization of the induction conditions

**Figure 2:**
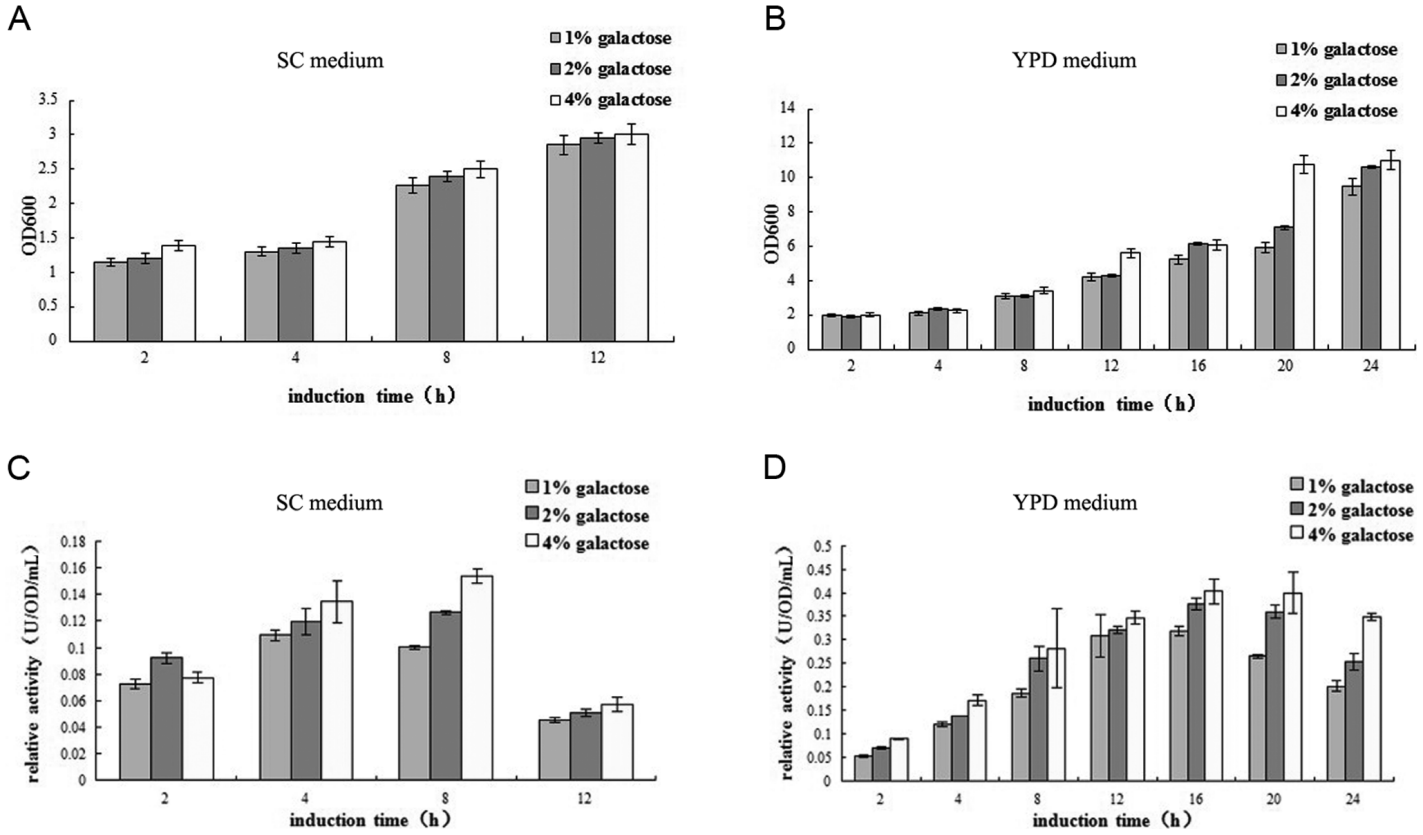
Analysis of the growth of yeast and the induced expression of AChE in different culture conditions. After induction in SC (A) and YPD (B) medium with 1%, 2%, or 4% of galactose for different time periods, the OD_600_ values of yeast suspension were determined. After induction in SC (C) and YPD (D) minimal medium with 1%, 2%, or 4% of galactose for different time periods, the cultures were harvested and the relative enzyme activity of induced AChE was determined. Error bars represent the standard deviation of mean values.

To explore the optimal induction conditions, we investigated the effects of induction time, galactose concentrations, and different culture mediums on induced DmAChE expression through an orthogonal test assay. Growth in YPD medium was generally higher than that in SC medium (Figure 2A and 2B). The expression level of DmAChE in YPD medium was also generally higher than that in SC minimal medium (Figure 2C and 2D). The expression level induced with 4% galactose was higher than with 2% and 1% galactose in both SC minimal medium and YPD medium. And the increasing tendency of expression became weak after 8 h. Considering that inducting for 12 h or 16 h was more difficult and that the DmAChE activities were not significantly higher than that of 8 h, we chose 4% galactose and 8 h induction time in YPD medium as the optimal induction condition.

### DmAChE inhibition studies

**Figure 3:**
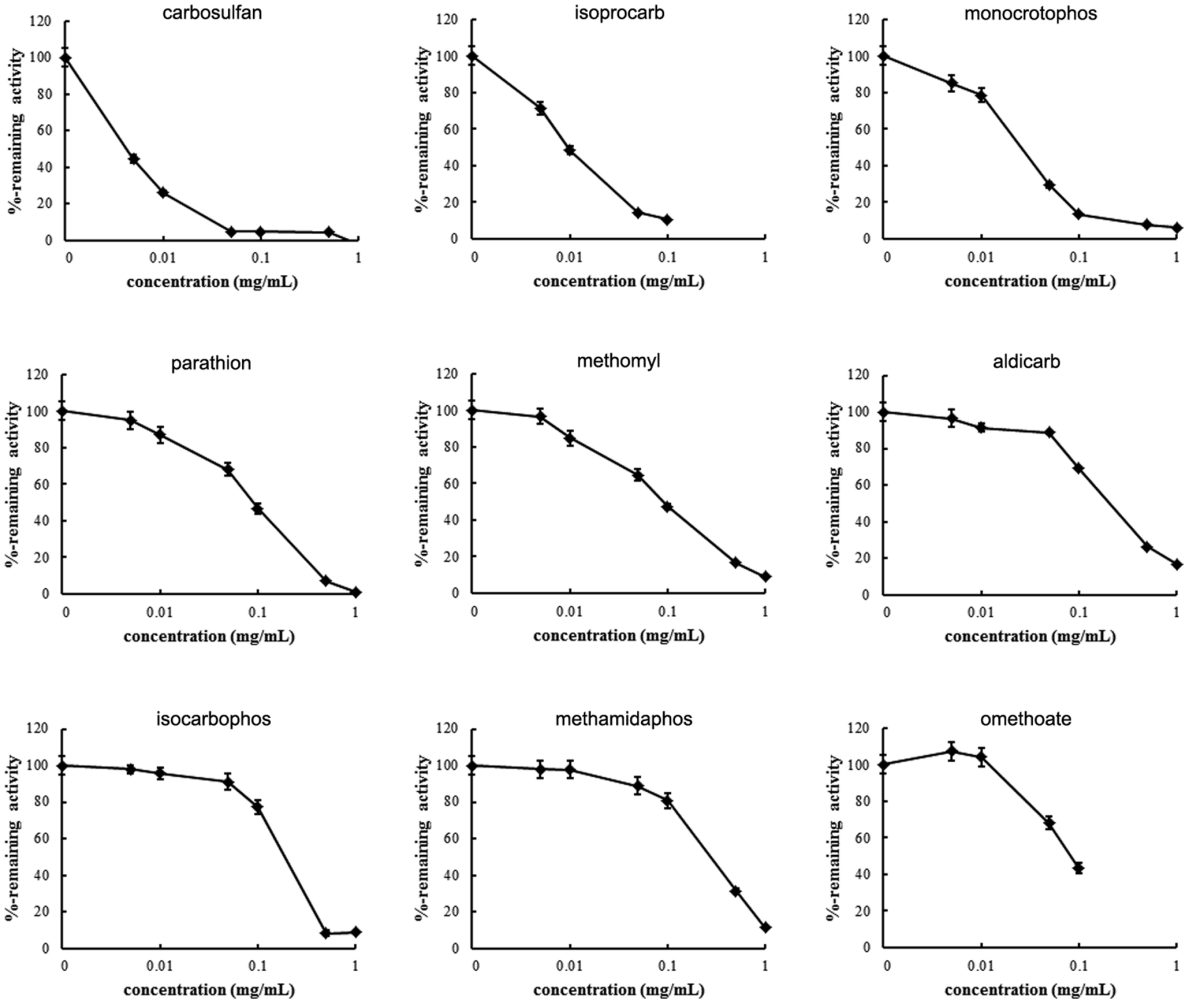
The inhibition curve of some organophosphorus and carbamate pesticides. The yeast with AChE expression were collected, re-suspended in PBS buffer and seeded in 96-well plate. After incubation with various concentrations of different pesticides, the remaining enzyme activities were determined. Error bars represent the standard deviation of mean values.

To investigate the potential application of DmAChE in OP and CB insecticide detection, we determined the sensitivity of DmAChE to each of nine types of insecticides according to GBT5009.199–2003, which is the national standard (Rapid determination for organophosphate and carbamate pesticide residues in vegetables) issued by the Standardization Administration of China, using *S. cerevisiae* suspension as the pure enzyme. And we found that the activity of DmAChE expressed on the surface of yeast significantly decreased after incubations with different pesticides (Figure 3). Although the inhibition effects varied in different pesticide, the enzyme showed good sensitivity to most insecticides. Six pesticides could inhibit the activity more than 50% at 0.1 mg/mL. Also, the inhibition effects of OP and CB insecticides showed in a concentration dependent manner (Figure 3). These results indicate that our expressed enzyme showed good sensitivity and can apply for the rapid determination for organophosphate and carbamate pesticide residues.

## Discussion

In this study, we have described the expression of *D. melanogaster* AChE carrying the secretion and glycosylphosphatidylinositol (GPI)-anchoring signals in *S. cerevisiae*. Induced *S. cerevisiae* could serve as an expression system for functional AChE for the detection of OP and CB insecticides with relatively low detection limits. Thus, this study shows great potential for the further development of rapid detection technologies.

In the optimization of induction conditions, we speculated that the DmAChE activity was associated directly to expression level on the surface. We also speculated that the growth conditions of *S. cerevisiae* itself could influence the expression level directly. As in Figure 2A, *S*. *cerevisiae* enter into the logarithmic phase about 6 h after induction followed by stationary phase and death phase as a result of nutrient depletion in SC minimal medium. In comparison, the stationary phase and death phase is delayed in YPD medium (Figure 2A and 2B). DmAChE activity is directly proportional to the concentration of the inductor galactose. An exception to this was seen with 4% galactose and 2 h induction in SC medium. We speculated that this result is due to the short induction time, which greatly weakens the influence of high concentrations of inductor.

Recombinant DmAChE has been previously produced in *Pichia pastoris* [28], [30]. In this study, the signal for the GPI anchor attachment was removed to induce DmAChE expression and secretion into the culture medium. However, the isolation and purification of recombinant DmAChE was costly. The yeast containing the surface enzyme could potentially be immobilized or used directly in insecticide detection. We compared our results with other reports using different enzymes. Firstly, our recombinant enzyme showed more sensitivity to carbaryl and carbofuran than natural AChE purified from different species including *Oncorhynchus tshawytscha*, *Clarias batrachus*, *Electrophorus electricus* and *Bos Taurus* [31], [32]. Secondly, compared to recombinant enzymes such as *Schizaphis graminum* AChE and *P. papatasi* AChE produced in different baculovirus-based insect cells expression systems, our yeast-expressed AChE could be more effectively inhibited by paraoxon and/or carbaryl [33], [34]. Even if the yeast-based recombinant *D. melanogaster* AChE expression system was also used, our surface display system showed more advantages and got better effects on monocrotophos and omethoate [30], [35], [36]. This might be due to the stability of the enzyme, in our system the yeast suspension was used directly, but not dried powder form in other studies. Our study provides for the possibility of the development of a biological sensor and other automatic detection techniques for a large number of agricultural products containing excess OP and CB insecticides. Through large scale fermentation, with omission of protein extraction and purification steps, the economic costs could decrease dramatically.

Compared to traditional chromatographic techniques, this system is more suitable for rapid detection strategies with excellent sensitivity. Other expression systems focus more on the yield and process of the recombinant enzyme [37]–[39] and less on the potential costs and time-consuming extraction and purification procedures after enzyme expression and secretion. There are also many studies regarding surface display systems, which we modified and applied to the current system of detection of OP and CB insecticides. As an immobilized carrier itself, yeast increases the stability of the enzyme and can be used directly as the functional enzyme in insecticide detection. Without the extraction and purification steps, the whole process from fermentation to acquisition takes 9 h. Also, the activity of DmAChE in the yeast suspension could be maintained at more than 90% following storage at 4°C for 6 months (data not shown), which fits the requirement of fast detection methods.

In summary, the recombinant enzyme DmAChE was expressed and anchored on the surface of *S. cerevisiae* with the yeast suspension itself used for the detection of OP and CB insecticides directly with high stability. This system offers a promising alternative for the rapid detection of insecticidal residues.

## Materials and Methods

### Strains and culture conditions

*S. cerevisiae* INVSci of yeast was purchased from Invitrogen (Grand Island, NY). Cells were grown in Yeast Extract Peptone Dextrose (YPD) medium (1% yeast extract, 2% peptone, 2% dextrose) or Synthetic Complete (SC) minimal medium (0.67% yeast nitrogen base, 2% carbon source, 0.01% of each adenine, arginine, cysteine, leucine, lysine, threonine, tryptophan, uracil, 0.005% of each aspartic acid, histidine, isoleucine, methionine, phenylalanine, proline, serine, tyrosine and valine). For selective media, uracil was omitted in SC. Competent *Escherichia coli* TOP10 and DH5α were purchased from Tiangen (Beijing, China). Ampicillin, when necessary, was added to a final concentration of 100 μg/mL.

### Chemicals

Acetylthiocholine iodide (ATChI), 5,5′-Dithiobis (2)-nitrobenzoic acid (DTNB), 1,5-Bis (4-allyldimethylammoniumphenyl) pentan-3-one dibromide (BW284c51) and tetraisopropyl pyrophosphoramide (iso-OMPA) were purchased from Sigma (St. Louis, MO). OP and CB insecticides (purity ≥97. 7%) were purchased from Chinese Academy of Agricultural Sciences (Beijing, China).

### Plasmid construction

**Figure 4:**
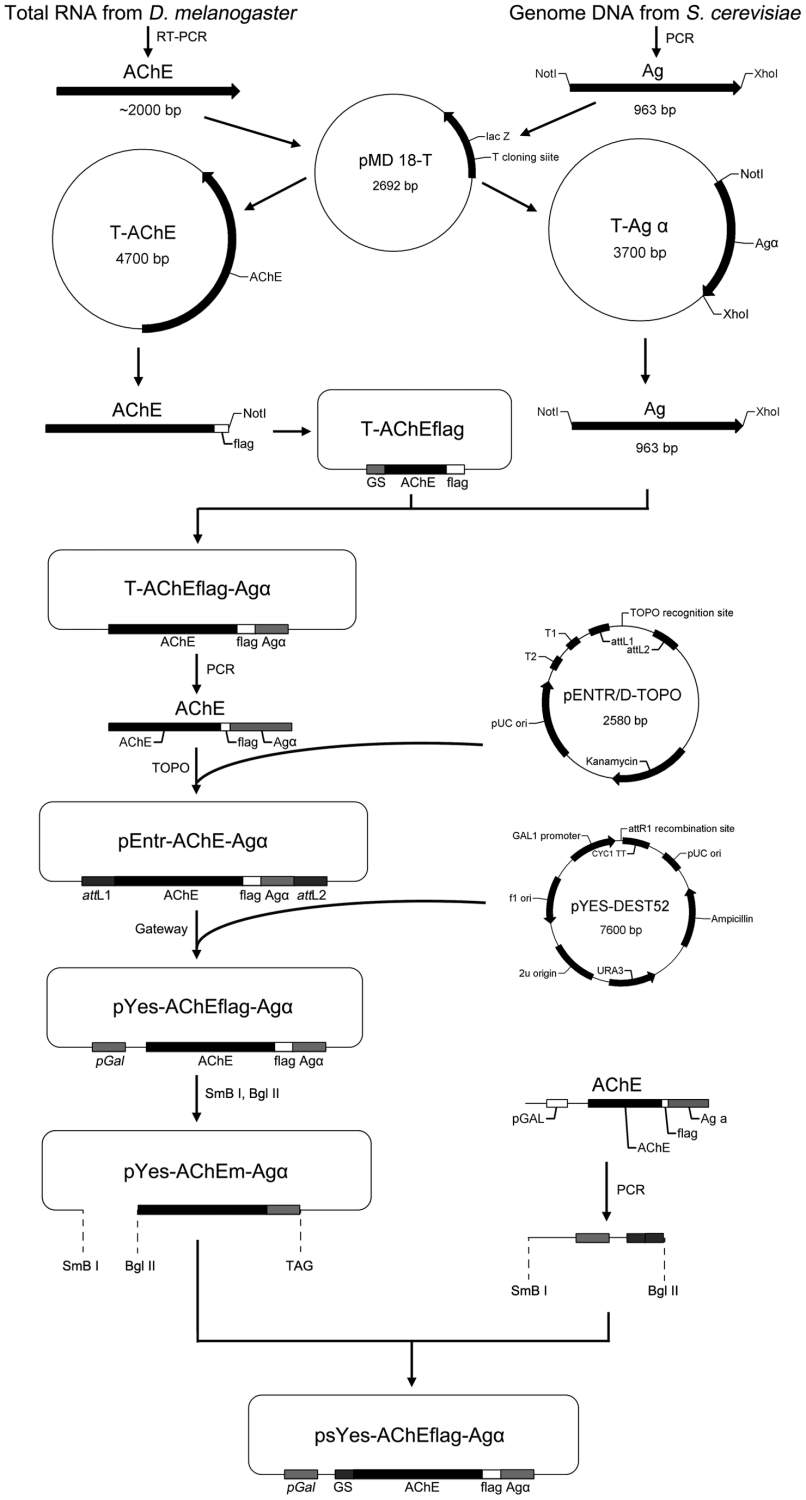
The construction of plasmid pYES-DEST52.

*D. melanogaster* was kindly provided by Dr Jiankang Liu (Institute for Nutritional Sciences, Shanghai Institute of Biological Science, Chinese Academy of Sciences). DmAChE (Genbank ID: NM_057605) and the 3′ half of the α-agglutinin (Agα) gene from *S. cerevisiae* was cloned into pMD18-T. The fragment DmAChE was amplified by PCR using primers AChE-F and AChE*flag*-R (all primers are listed in Table 1), adding a FLAG fragment. The amplified fragment was then re-inserted into pMD18-T. A 960-bp *Not*I-*Xho*I fragment containing the Agα gene was subsequently ligated into this construct. The target fragment was amplified by PCR using primers EntR-F and EntR-R and inserted into pENTR™ Directional TOPO vector (Invitrogen), which was subsequently recombined with pYes-DEST52 (Invitrogen). In order to replace the 114-bp secretion region of DmAChE with the 75-bp glucoamylase secretion peptide, a 1.4-kb fragment named GSU was amplified by PCR using primers pGSU-F1 and pGSU-R. A 0.4-kb fragment named GSD2 was amplified by PCR using primers pGSD-F2 and pGSD-R with GSD1 (amplified by pGSD-F1 and pGSD-R) as template. GSU and GSD2 was ligated by overlap PCR (5 μL 10×KOD buffer, 4 μL 2 mM dNTPs, 2 μL 25 mM MgCl_2_, 1 μL KOD plus, 2 μL equal molar GSU and GSD2 as template, 48 μL in total; 94°C, 3 min; 94°C, 30 s; 56°C, 30 s; 68°C, 90 s for 5 cycles followed by the addition of 1 μL 20 mM pGSU-F1/pGSD-R followed by cycles). The resultant fragment was excised by *SnaB*I-*Bgl*II and ligated into pYes-DEST52-AChE for the final construct. The whole construction process is shown in Figure 4.

**Table 1:** Primers used in this study.

Primer name	sequence (5′ to 3′)
AChEF1	CATATGATGGCCATCTCCTGTCGGCAGAG
AChER1	CTCGAGGAAAACCCTTTTGGTTCGCAATGCGGC
AChE-F	ATGGCCATCTCCTGTCGG
AChE*flag*-R	GCGGCCGCCTTGTCATCGTCATCTTTATAATCACTTCCCGAA
	TCGCCATC
Aga-F	GCGGCCGCTAGCGCCAAAAGCTCTTTTATCT
Aga-R	CTCGAGCTAGAATAGCAGGTACGACAAAAGC
EntR-F	CACCATGGCCATCTCCTGTCGG
EntR-R	TAGAATAGCAGGTACGACAAAAGC
pGSU-F1	TGCGGGTGCATTTTTTCAAGATAAAG
pGSU-R	GAGAAAGAATGAAACTTTCAATGGCAAATTGAACAGTTGC
	ATGGTGAAGGGGGCGGC
pGSD-F1	TTCTTTCTCGTCCTCTCTTACTTTTCTTTGCTCGTTTCTGCTG
	TCATCGATCGCCTGG
pGSD-F2	ATGCAACTGTTCAATTTGCCATTGAAAGTTTCATTCTTTCTC
	GTCCTCTCTTACTTTTC
pGSD-R	GACACGTTGGTGTTGGGGTTCC

### Yeast transformation

The small scale transformation of *S. cerevisiae* was conducted using S.c.EasyComp. Transformation Kit (Invitrogen) as per the manufacturer’s instructions.

### The induction of DmAChE expression

The clones expressing pYES-DEST52-AChE were selected and cultured in medium supplemented with 2% glucose at 30°C and 300 rpm. The OD_600_ values were adjusted to 0.4 with fresh induction medium. Under the GAL1 promoter, the transcription of recombinant DmAChE was induced through the removal of glucose and the addition of galactose as the sole carbon source.

### Orthogonal test

In the orthogonal test, YPD medium and SC minimal medium with 1%, 2% and 4% galactose were used. After specific periods of induction (2, 4, 8, 12 and 24 h), cultures were harvested and centrifuged at 1,500 rcf for 5 min at 4°C. The cells were re-suspended and immediately used for activity and inhibition assays to determine growth rate comparisons and the effect of DmAChE expression and display.

### DmAChE activity assay

DmAChE activity was assayed by the method reported previously [40] using 2 mM final concentration of acetylthiocholine iodide as substrate and 2 mM final concentration of DTNB as the chromogenic agent. The *S. cerevisiae* suspension (OD_600_ = 1.0) served as the active enzyme. The OD_412_ values were determined once every 0.5 min for 15 min in 37°C. The slope of the linear of absorbance versus time reflects the catalytic activity. The relative enzyme activity was acquired from the standard curve based on the results of commercial pure AChE enzyme. OP and CB insecticide stocks were prepared in acetone and diluted with phosphate buffered saline (PBS) buffer (pH 7.6). For each sample, 10 μL pesticide solution was pre-incubated with yeast suspension for 15 min at 37°C. Uninduced *S. cerevisiae* (without expression of DmAChE on the surface) served as the negative control, whereas *S. cerevisiae* without pesticide incubation served as the positive control. The activity of the positive control was considered 100%. The remaining activity of DmAChE is reflected as the slope of the following equation:
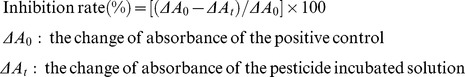
When the inhibition rate ≥50%, the pesticide was considered to be detected.
