# Correction: Surface Display of Recombinant *Drosophila melanogaster* Acetylcholinesterase for Detection of Organic Phosphorus and Carbamate Pesticides

**DOI:** 10.1371/annotation/5c68b3d3-240f-4e87-b394-ca5301479cef

**Published:** 2013-10-08

**Authors:** Jingquan Li, Qian Ba, Jun Yin, Songjie Wu, Fangfang Zhuan, Songci Xu, Junyang Li, Joelle K. Salazar, Wei Zhang, Hui Wang

As a result of errors in the typesetting process, the citation was incorrect. The correct version of the citation is available below.

Li J, Ba Q, Yin J, Wu S, Zhuan F, et al. (2013) Surface Display of Recombinant *Drosophila melanogaster* Acetylcholinesterase for Detection of Organic Phosphorus and Carbamate Pesticides. PLoS ONE 8(9): e72986. doi:10.1371/journal.pone.0072986 

